# The Motility and Mesenchymal Features of Breast Cancer Cells Correlate with the Levels and Intracellular Localization of Transglutaminase Type 2

**DOI:** 10.3390/cells10113059

**Published:** 2021-11-06

**Authors:** Nicoletta Bianchi, Federica Brugnoli, Silvia Grassilli, Karine Bourgeois, Jeffrey W. Keillor, Carlo M. Bergamini, Gianluca Aguiari, Stefano Volinia, Valeria Bertagnolo

**Affiliations:** 1 Department of Translational Medicine, University of Ferrara, 44121 Ferrara, FE, Italy; federica.brugnoli@unife.it (F.B.); grsslv@unife.it (S.G.); s.volinia@unife.it (S.V.); valeria.bertagnolo@unife.it (V.B.); 2 Laboratory for Advanced Therapy Technologies (LTTA), 44121 Ferrara, FE, Italy; 3 Department of Chemistry and Biomolecular Sciences, University of Ottawa, Ottawa, ON K1N 6N5, Canada; bourgeois.e.k@gmail.com (K.B.); jkeillor@uottawa.ca (J.W.K.); 4 Department of Neuroscience and Rehabilitation, University of Ferrara, 44121 Ferrara, FE, Italy; carlo.bergamini@unife.it (C.M.B.); gianluca.aguiari@unife.it (G.A.)

**Keywords:** transglutaminase type 2, breast cancer, motility, EMT, NC9

## Abstract

We have investigated motility in breast cancer cell lines in association with the expression of Transglutaminase type 2 (TG2) as well as upon the administration of Doxorubicin (Dox), an active cytotoxic agent that is employed in chemotherapy. The exposure of MCF-7 cells to the drug increased TG2 levels, triggering epithelial–mesenchymal transition (EMT), thereby supporting cell motility. The effects of Dox on the movement of MCF-7 cells were counteracted by treatment with NC9, a TG2 inhibitor, which induced morphological changes and also reduced the migration of MDA-MB-231 cells exhibiting high levels of TG2. The physical association of TG2 with the cytoskeletal component vimentin appeared pivotal both in drug-treated MCF-7 and in MDA-MB-231 cells and seemed to be independent of the catalytic activity of TG2. NC9 altered the subcellular distribution of TG2 and, consequently, the co-localization of TG2 with vimentin. Furthermore, NC9 induced a nuclear accumulation of TG2 as a prelude to TG2-dependent gene expression modifications. Since enzyme activity can affect both motility and nuclear functions, targeting of this protein could represent a method to improve therapeutic interventions in breast tumors, particularly those to control progression and to limit drug resistance.

## 1. Introduction

Breast cancer (BrCa) represents a treatable tumor, although many phenotypes still escape therapeutic control with relapses and aggressive features in correlation with histopathological grading. In many tumors, the expression of TG2 is strongly associated with the ability of cancer cells to change their phenotype, leading to EMT, which represents a step favoring migration from the primary lesion and the seeding of metastases [1]. Confirming its role in tumor progression, TG2 inhibitors have been employed to reverse the migration/invasion hallmarks of skin cancer [2].

These malignancy-related phenomena are sustained by TG2 through transamidation and GTPase activities that interconnect signaling inflammatory cascades, such as the TG2/Snail/E-cadherin axis that is mediated by Reactive Oxygen Species through Transforming Growth Factor β1 [3], and the Nuclear Factor-kappa B (NF-κB). NF-κB forms a positive feedback loop with TG2 that is responsible for the overexpression of TG2 [4]. In this context, the triple-negative phenotype MDA-MB-231 cells highly express the enzyme because the *TGM2* gene promoter is completely demethylated [5]. In these tumor cells, the transcriptional regulation of TG2 is under the control of the NF-ĸB signaling pathway [6,7,8], which is strongly associated with their proliferative and infiltrating capacities [9]. In contrast, breast tumor cells with an ER positive phenotype, such as MCF-7 cells, express TG2 at low levels, although these levels are still high enough to trigger EMT via Snail, Twist, and Hippo. This pathway leads to a decrease of the epithelial marker E-cadherin and increases the mesenchymal marker vimentin [10], which belongs to the pattern of genes that is dysregulated by Snail in cells that are resistant to several drugs, such as Paclitaxel, Docetaxel, and Dox [11]. As this network is strongly interconnected to TG2 [3], the levels of this enzyme appear to be much higher in cervical cancer HeLa cells after treatment with Dox [12].

During EMT, epithelial cancer cells lose their cell-to-cell connections and undergo modifications in shape and cytoskeletal organization, acquiring mesenchymal characteristics. In the organization of the cytoskeleton, it has long been known that TG2 influences actin and tubulin polymerization and associates with vimentin in the intermediate filaments [13]. More recent reports describe the involvement of TG2 in chromatin dynamism through actin remodeling and the aberrant reorganization of the cytoskeleton [14,15]. As these events orchestrate a flow of processes that imply changes in cell polarity and motility, improving migration performance, the identification of the main players is a critical point for the control of tumor invasiveness and metastasis [16,17].

On these premises, we aimed to investigate the relationships between TG2 and motility in breast tumor cells in greater detail, focusing on vimentin, which represents an important element of the filamentous cytoskeleton and a pivotal marker of EMT [18]. Although the interactions between these proteins have already been described in other cellular models, indicating that vimentin is a target protein of the transamidase activity of TG2 [19,20], no experiments have investigated a direct interaction in BrCa. In addition, many findings have demonstrated a correlation of their expression, especially in relation to exposure to anticancer drugs and/or EMT [21], and relevant results have focused on their role in the extracellular matrix [22,23]. We believe that interactions between TG2 and vimentin could be possible and crucial, even inside the cell, especially if we consider the overlap of their localization and relationships with filaments that have been widely reported upon in the literature [24,25,26,27] in addition to the link with EMT [28,29].

In the last few years, the use of specific TG2 enzymatic activity inhibitors has substantially contributed to the establishment of a role of TG2 in cell motility. It has been reported that the NC9 inhibitor blocks all stages of osteoclastogenesis, in particular pre-osteoclast migration, and affects podosome belt formation in osteoclasts, in which TG2 co-localizes with other members of tranglutaminase family, such as TG1 and FXIIIa [30]. With a similar approach, we have treated MCF-7 cells where the low level of TG2 can be up-modulated by Dox [21,22] and MDA-MB-231 cells showing high basal levels of TG2 with the NC9 inhibitor and investigated the effects on motility, revealing an unprecedented role of TG2 in interacting with vimentin in breast tumor cells.

## 2. Materials and Methods

### 2.1. Cell Cultures and Treatments

MCF-7 (ER^+^/PR^+^/HER2^−^) and MDA-MB-231 (ER^−^/PR^−^/HER2^−^) cells were purchased from the American Type Culture Collection (Rockville, MD, USA) and were grown in Dulbecco’s modified Eagle’s medium (DMEM, Gibco Laboratories, New York, NY, USA) supplemented with 10% fetal bovine serum (FBS, Gibco Laboratories, New York, NY, USA) and 50 U/mL penicillin plus 50 μg/mL streptomycin (Gibco Laboratories, New York, NY, USA). The cultures of both cell lines were performed at 37 °C and 5% CO_2_ and were treated with the drugs when they reached the 70% confluence.

Dox (Merck Life Science, Milan, Italy) was added at concentration of 2 μM, and treatment was carried out for up to 72 h. The compound NC9 is *N*-α-carbobenzyloxy-*N*-ε-acryloyl-l-lysine(2-(2-dansylaminoethoxy)-ethoxy)ethanamide, an irreversible inhibitor that blocks both transamidation and GTPase activities. It was synthetized in the Keillor lab [31], but it is also commercially available from Zedira (Darmstadt, Germany). It was added to the cultures at the final concentration of 30 μM for 16 h [32,33,34,35]. In the case of combined treatment with Dox, the inhibitor was added after 20 h of pre-exposure to Dox.

In all of the experimental conditions, the cell viability was checked with the Trypan Blue Exclusion Test, and the morphology was analyzed with a phase contrast microscope (Diaphot, Nikon, Melville, NY, USA).

### 2.2. Parallel Artificial Membrane Permeability Assay (PAMPA)

The cell permeance of NC9 was estimated by two different PAMPA evaluations. In the first evaluation, an artificial hexadecane membrane was prepared by adding a solution of 5% hexadecane in hexane to the membrane of a filter plate. The plate was then dried at room temperature for 1 h, allowing the hexane to evaporate. Phosphate-buffered saline (PBS, pH = 7.4) was added to the lower acceptor chamber, and a solution of NC9 in 5% DMSO in the same buffer was added to the upper donor chamber. After incubation with gentle shaking (75 rpm) for 5 h at 37 °C, the concentrations of NC9 in the upper and lower chambers were determined by HPLC-UV. The effective permeability, in units of cm/s, was determined to be Log P_e_ = −5.26 ± 0.01, following measurements in triplicate and calculation using the following equation [36,37]:

$$Log\;P_{e}=Log\left\{C\times \left[-ln\left(1-\frac{{\left[\mathrm{NC}9\right]}_{\mathrm{acceptor}}}{{\left[\mathrm{NC}9\right]}_{\mathrm{equilibrium}}}\right)\right]\right\}$$

where $C=\frac{volume_{\mathrm{donor}}\times volume_{\mathrm{acceptor}}}{\left(volume_{\mathrm{donor}}+volume_{\mathrm{acceptor}}\right)\times Area\times Time}$.

The second evaluation was performed by Pharmaron Inc. (Louisville, KY, USA), according to the following general protocol: An artificial membrane was prepared by adding a solution of 1.8% lecithin in dodecane to the membrane of a filter plate. Phosphate-buffered saline (PBS, pH = 7.4) was added to the lower chamber, and NC9 in PBS (0.1% DMSO) was added to the upper chamber. After incubation at 25 °C with gentle shaking (60 rpm) for 16 h, the concentration of NC9 in the upper and lower chambers was determined by HPLC-MS. Measurements were performed in triplicate using the same equation shown above [38].

### 2.3. Reverse Transcription and Real Time Quantitative PCR

Samples derived from untreated or treated cells were processed to purify the RNA using TRI Reagent^®^ (Merck Life Science), as described in the manufacturer’s protocols, and 1 µg of total RNA was reverse transcribed with TaqMan^®^ Reverse Transcription Reagents kit (Thermo Fisher Scientific, Waltham, MA, USA). Quantitative PCR (qPCR) reactions to detect TG2 mRNA in the untreated and treated samples were performed with primers and under the conditions reported by Franzese et al. [39]. For quantification, the hypoxanthine phosphoribosyl transferase 1 (HPRT1) gene was employed as a housekeeping gene [40], and the relative fold change of expression of the target gene was calculated with respect to the sample that was only treated with the vehicle, according to the formula 2^−ΔΔCT^.

### 2.4. Nuclear Protein Extracion, Immunoprecipitation and Immunochemical Analysis

The preparation of the nuclear extracts was performed from 15 × 10^6^ MDA-MB-231 cells grown adherently in T175 flasks and that had been cultured at a confluence of about 70% in the different experimental conditions. After three washes in PBS, nuclear pellets were resuspended in a solution comprising 2 mL PBS plus 2 mL nuclear isolation buffer (1.28 M sucrose, 40 mM Tris-HCl pH = 7.5, 20 mM MgCl_2_, 4% (v/v) Triton X-100) and 6 mL H_2_O and were incubated on ice for 20 min before being collected by centrifugation at 2500× *g* for 15 min at 4 °C. These nuclear pellets contained nuclear membranes and DNA and were denatured and analyzed by Western blotting; a second type of samples consisted of the soluble phase of the nuclear extracts prepared in parallel experiments as follows: the nuclei were resuspended in 200 µL freshly prepared ice-cold buffer containing 25 mM Tris Ph = 7.5, 150 mM KCl, 5 mM EDTA, 0.5 mM DTT, 0.5% NP40, plus HALT protease inhibitor cocktail 1× from Thermo Fisher Scientific, Italy, and were sheared on ice by passage through a 27 gauge needle with 15–20 strokes, following the protocol reported by Franzese et al. 2019 [39]. These nuclear lysates constituted the soluble phase and were obtained as supernatants by removing nuclear membranes, DNA, and debris by centrifugation at 16,000× *g* for 10 min at 4 °C.

Immunoprecipitation experiments of whole-cell extract from MCF-7 and MDA-MB-231 cells cultured in the presence or absence of drugs were carried out with antibodies against TG2 (Ig036, Zedira, Darmstadt, Germany), vimentin (sc-7558, Santa Cruz Biotechnology), or with a non-specific IgG, which was used as a negative control (sc-53344, Santa Cruz Biotechnology), and precipitated with protein A-Sepharose (Pharmacia, Uppsala, Sweden), as previously described by Bertagnolo et al. [41].

Nuclear pellets, nuclear lysates in soluble phase, cellular lysates, and immunoprecipitates were run with 7.5% denaturing polyacrylamide gel and were transferred to nitrocellulose membranes (GE Healthcare Life Science, Milan, Italy), which were then stained with specific antibodies to detect TG2 (Ig036, Zedira, Germany), E-cadherin (sc-7870), vimentin (sc-7558), Sp1 (sc-14027 X), RARα (sc-551), EIF3β (sc-271539), and GAPDH (sc-47724) from Santa Cruz Biotechnology; N-cadherin (MA1-2002) and MAX (PA5-29745) from Thermo Fischer Scientific (Rockford, IL, USA); c-Myb (orb256699, Biorbyt, San Francisco, CA, USA); RXRα (ab125001, Abcam, Cambridge, UK); YY1 (ab245365, Abcam); and Actin (A4700) and β-Tubulin (T4026) (Merck Life Science), as previously reported [42].

We used the ECL system (Perkin-Elmer, Boston, MA, USA) for the detection of immune complexes by chemiluminescence. The chemiluminescence patterns were acquired with the ImageQuantTM LAS 4000 instrument (GE Healthcare Life Science, Milan, Italy), a CCD-imager equipped with a 16-bit, 3.2 Mpixel camera fitted with a large aperture F0.85 lens. This system, which is able to distinguish more than 65,000 different gray levels, automatically determines the optimal exposure time to visualize the protein bands of both high and low intensity at the same time without risking signal weakness or saturation. After image capture, chemiluminescence was quantified by means of the Image Quant TL (GE Healthcare Life Science, Milan, Italy) software.

### 2.5. Real-Time Assays of Cell Migration and Invasion

Cells under different experimental conditions were collected, and their migration and invasion capabilities were assayed by xCELLigence RTCA system (Real-Time Cell Analyzer System, Acea Biosciences Inc., San Diego, CA, USA) [43]. For both assays, 4 × 10^5^ cells/well were posed onto the top chambers of CIM-16 plates. For the analysis of invasiveness, the upper chamber was layered with diluted Matrigel (1:20 and 1:40 in serum-free medium, BD Biosciences, Milan, Italy), and the bottom chamber was filled with medium containing 5% FBS as a chemoattractant in the migration assay or 10% FBS in the invasiveness assay. Each determination was performed in quadruplicate, with signal detection every 15 min for 24 h. Impedance values were expressed as a dimensionless parameter (Cell Index, CI), and values greater than 0.1 were considered positive. The rate of cell migration was also quantified by calculating the slope indicating the steepness, inclination, gradient, and changing rate of the CI curves over time.

### 2.6. Immunocytochemical and Confocal Analysis

For the immunocytochemical analysis, cells grown on glass slides were fixed with freshly prepared 4% paraformaldehyde. To analyze vimentin expression, the cells were reacted with the specific primary antibodies for 3 h at room temperature in Net Gel solution (150 mM NaCl, 5 mM EDTA, 50 mM Tris-HCl pH 7.4, 0.05% NP40, 0.25% Carrageenan Lambda gelatin, and 0.02% Na azide (all from Merck Life Science). Samples were then labeled with FITC-conjugated secondary antibodies (Thermo Fisher Scientific) at room temperature in the dark, as reported by Brugnoli et al. [44].

To analyze the TG2/vimentin co-localization, cells were fixed stained with both primary antibodies for 3 h in Net Gel and were further labeled with both FITC- and TRITC-conjugated secondary antibodies (Thermo Fisher Scientific) at room temperature in the dark [44]. After being washed, all samples were incubated with 0.5 µg/mL 4′,6-diamidino-2-phenylindole (DAPI) (Merck Life Science), dried with ethanol, and mounted in glycerol containing 1,4-diazabicyclo [2.2.2] octane (DABCO) (Merck Life Science) to delay fading. A Nikon Ci-L microscope (Nikon, Melville, NY, USA) was employed to analyze the fluorescent samples, and NIS-ELEMENTS D software was used for a DS-Qi2Mc digital camera that was used to acquire images (Nikon, Melville, NY, USA).

To analyze TG2 distribution using confocal microscopy, the samples were labelled with the secondary antibody, incubated with DRAQ5 Stain (Cell Signalling Technology, Leiden, The Netherlands) for half an hour at 37 °C, and mounted in glycerol containing DABCO. A Zeiss LSM 50 laser scanning confocal microscope, equipped with a 63× oil immersion Plan-Neofluar objective (Carl Zeiss, Göttingen, Germany), was used to obtain the images. To measure vimentin staining, TG2/vimentin co-localization, and TG2 nuclear staining, digitized images were analyzed with the ImageJ software (http://rsb.info.nih.gov/ij/, accessed on 1 October 2021), and fluorescence values of integrated density (IntDen) of at least 20 cells from three different areas were expressed as arbitrary units/cell.

### 2.7. Statistical Analysis

The results were represented as the average  ±  the standard deviations of three independent experiments. Statistical analysis was performed by two-tailed Student’s *t* test for unpaired data using the GraphPad Prism 6.0 statistical package (GraphPad Software, San Diego, CA, USA). We considered *p* values < 0.05 to be statistically significant.

## 3. Results

### 3.1. Dox-Induced Changes in Morphology, Motility and EMT Markers Involve TG2 in MCF-7 Cells

**Figure 1:**
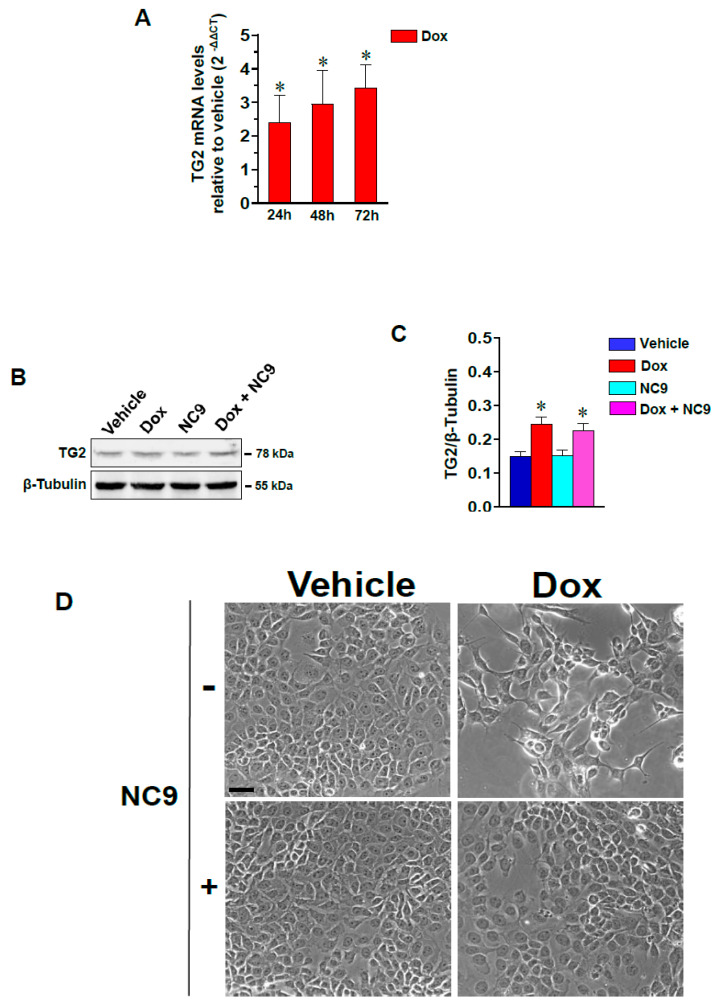
(**A**) RT-qPCR analysis of TG2 mRNA in MCF-7 cells treated with Dox for the indicated time (h). Relative transcript levels were determined using the 2^−ΔΔCT^ method and were reported as fold changes with respect to cells cultured in the presence of DMSO (vehicle). (**B**) Representative immunoblot analysis performed with the indicated antibodies on lysates from MCF-7 cells cultured in the presence of vehicle or Dox with or without the addition of the TG2 inhibitor NC9. (**C**) Levels of TG2 as deduced from the chemiluminescence signal normalized with β-Tubulin, which was used as an internal control for the equivalence of loaded proteins. The data are shown as the mean of three separate experiments performed in triplicate ± SD. * *p* < 0.05 versus vehicle. (**D**) Representative phase-contrast images of MCF-7 cells growing on glass dishes in the presence of vehicle or Dox with or without NC9. Bar = 50 µm.

Since high levels of TG2 are reported in MCF-7 cells that are resistant to Dox [22], we evaluated the expression of TG2 as a function of the time of treatment with the drug. As shown in Figure 1A, the fold change of TG2 mRNA was 2.4 ± 1.8 already at 24 h of exposure (quantified by RT-qPCR with respect to control treated only with vehicle and taken as 1), which represents an early drug-mediated cell response that leads to fold increases of 3.4 ± 1.9 at 72 h. The changes observed at the early time of Dox administration reflected those revealed by Western blot analysis, showing that the protein accumulated slightly in the samples that had been treated for 24 h (Figure 1B, 1C).

To assess the effects of Dox on the morphology of MCF-7 cells, we performed phase contrast microscopy analysis and revealed that the monolayers of the adherent cuboidal cells shifted to a pattern of spindle-like cells that were well separated from each other in the presence of Dox (Figure 1D). The relevance of TG2 in these morphological changes was investigated by conducting experiments on the cells treated with a combination of Dox and NC9, a cell permeant inhibitor. In this regard, measurements were performed using the equation reported in the Material and Methods section, and Log P_e_ was determined to be −5.06 ± 0.05. The values of P_e_ measured for the passive diffusion of NC9 across two different artificial membranes were both greater than 10^−6^ cm/s; consequently, NC9 can be considered to have at least moderate permeability.

Treatment with NC9 did not alter the levels of TG2 (Figure 1B,C), while as shown in Figure 1D, the morphological alterations induced by Dox were strongly reverted by NC9, suggesting the involvement of TG2 in the precocious effects of the drug and not only as part of late drug-resistance events.

**Figure 2:**
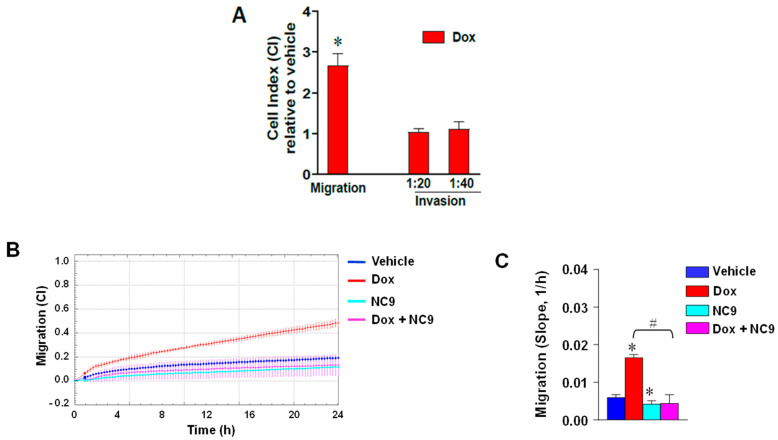
(**A**) xCELLigence-driven dynamic monitoring of migration and invasion through diluted matrigel (1:20 and 1:40) of MCF-7 cells cultured in the presence or absence of Dox for 36 h. Cell index (CI) values are reported relative to vehicle. (**B**) Dynamic monitoring of migration of MCF-7 cells cultured in the presence of Dox and/or NC9. Data are reported as CI ± SD. (**C**) The correspondent slope analysis that describes the steepness, inclination, gradient, and changing rate of the CI curves over time is shown. All of the data are shown as the mean of three independent experiments performed in triplicate ± SD. * *p* < 0.05 versus vehicle; # *p* < 0.05 between bars.

As cell elongation induced by Dox suggests modification in adhesion and cell motility, we measured cell movement by means of the xCELLigence RTCA system. As shown in Figure 2A–C, treatment with Dox induced a significant increase of the chemoactractive migration of the MCF-7 cells, which was completely counteracted by addition of NC9 (Figure 2B,C), confirming the involvement of TG2 in supporting cell motility. In this cell model, Dox failed to induce an increase of the invasion capability, which was explored using two concentrations of matrigel (Figure 2A).

**Figure 3:**
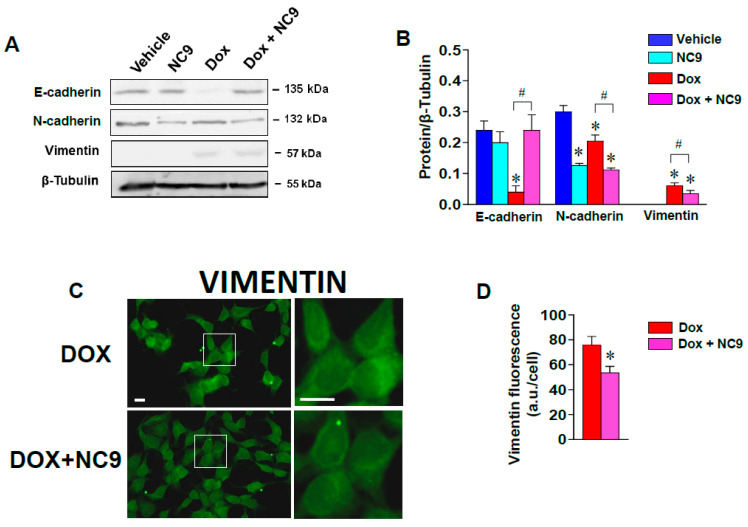
(**A**) Representative immunoblot analysis performed with the indicated antibodies on lysates from MCF-7 cells cultured in the presence of Dox and/or NC9. (**B**) Levels of E-cadherin, N-cadherin, and vimentin as deduced from the analysis of chemiluminescence, normalized with β-Tubulin, which was used as an internal control for the equivalence of the loaded proteins. The data are shown as the mean of three separate experiments performed in triplicate ± SD. * *p* < 0.05 versus vehicle; # *p* < 0.05 between bars. (**C**) Representative florescence images of MCF-7 cells grown on glass dishes for 36 h in the presence of Dox ± NC9 and then subjected to immunocytochemical analysis with the anti-vimentin antibody. The regions in the boxes are shown enlarged on the right. Bar: 20 µm. (**D**) Fluorescence intensity of digitized images calculated by the ImageJ software and reported as arbitrary units for cell (a.u./cell). * *p* < 0.05 versus Dox.

Since morphological changes are frequently associated with EMT and since Dox drives a shift towards a mesenchymal phenotype in MCF-7 cells [11], we analyzed the involvement of TG2 in motility events, looking at the expression of epithelial (E-cadherin) and mesenchymal (N-cadherin, vimentin) markers. As shown in Figure 3A,B, exposure to Dox largely decreased the amount of E-cadherin and induced the expression of vimentin, and both changes were reverted by administration of NC9 in combination with Dox, which also induced a decrease of N-cadherin. In addition, the exact quantification determined using the of ImageQuantTM LAS 4000 imager to acquire chemiluminescence signals also showed a low but significant decrease in vimentin (Figure 3A,B). To check whether the cellular content of vimentin could be modified in combined treatment, we performed immunofluorescence analysis on the MCF-7 cells exposed to Dox and/or to NC9. The images reported in Figure 3C confirmed that NC9 decreased the signal intensity of vimentin (as quantified Figure 3D). Note that vimentin presented perinuclear aggregation in correlation with cell adhesion, as reported by Kim et al. [29].

### 3.2. Impacts of TG2 on Cytoskeleton through Interactions with Vimentin and on Transcriptional Control in the Nucleus

**Figure 4:**
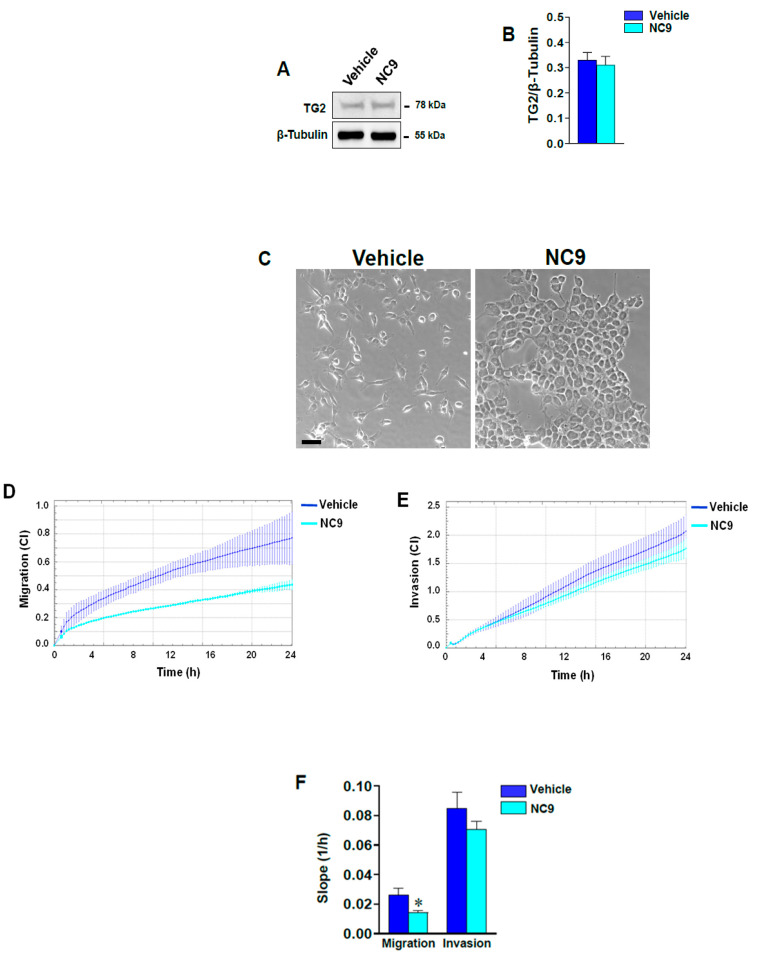
(**A**) Representative immunoblot analysis performed with the indicated antibodies on lysates from MDA-MB-231 cells cultured in the presence of vehicle or NC9. (**B**) Levels of TG2 as deduced from the analysis of chemiluminescence and normalized with β-Tubulin. (**C**) Representative phase-contrast images of MDA-MB-231 cells growing on glass dishes under the above reported conditions. Bar = 50 µm. (**D**,**E**) Dynamic monitoring of migration and invasion, respectively, of MDA-MB-231 cells under the same experimental conditions. Cell index mean ± SD is reported, and the corresponding slope analysis is shown in (**F**). The data show the mean of three separate experiments performed in triplicate ± SD. * *p*< 0.05 versus vehicle.

To further explore the role of TG2 on motility, we employed the NC9 inhibitor to treat mesenchymal-like MDA-MB-231 cells that were expressing high basal levels of TG2 (as compared to MCF-7 cells), representing a breast tumor model with more advanced malignant features. We found that although TG2 levels were not modified (Figure 4A,B), the cell morphology was altered in the presence of NC9 (Figure 4C), with reduction in the number of elongated MDA-MB-231 cells in favor of polygonal cells, which are closely associated with each other. Upon verification as to whether these changes reflected any alterations in cell motility, we observed a strong migration reduction of the MDA-MB-231 cells upon the addition of NC9, without any significant modification of invasive capabilities (Figure 4D–F). These data suggest that in BrCa cells, TG2 activity is engaged in cell motility rather than in the alteration of invasive potential.

**Figure 5:**
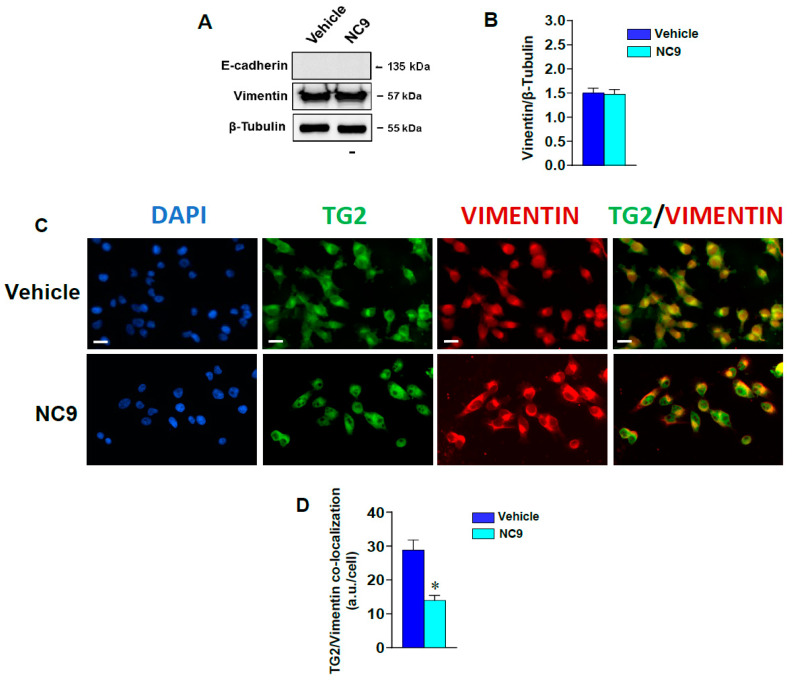
(**A**) Representative immunoblot analysis of E-cadherin and vimentin on total lysates from MDA-MB-231 cells grown in the presence of vehicle or NC9. (**B**) Levels of proteins deduced from the analysis of chemiluminescence. β-Tubulin was used as internal control for equivalence of proteins load. (**C**) Representative fluorescence images of MDA-MB-231 cells grown on glass dishes in the presence of vehicle or of NC9 and subjected to analysis of both TG2 and vimentin with a FITC- and a TRITC-coupled antibodies, respectively. DAPI data were also reported. Bar = 50 μm. (**D**) Fluorescence intensity of the TG2/vimentin merged staining (yellow) calculated by the ImageJ software and reported as arbitrary units for cell (a.u./cell) ± SD. * *p* < 0.05 versus vehicle.

We also compared the effects obtained after exposure to NC9 of MCF-7 cells treated with Dox (as an inducer of TG2) and of MDA-MB-231 cells, with respect to the expression of EMT markers. As shown in Figure 5A,B, in MDA-MB-231 cells, the vimentin levels (already elevated) were not significantly increased by NC9, which failed to induce the epithelial marker E-cadherin (not expressed in this type of cells). Additionally, we investigated the physical interaction between TG2 and vimentin on the basis of the TG2/vimentin association found in other cell models [19,20]. The immunocytochemical analysis reported in Figure 5C shows how in the control MDA-MB-231 cells, TG2 was mainly localized in the cytoplasm and was closely associated with the nucleus, and this distribution was substantially modified by NC9, which induced an apparent accumulation of the enzyme at the nuclear level. The fluorescence signals of TG2 and vimentin revealed that in untreated conditions, they co-localized specifically in the perinuclear zone. In contrast, NC9 caused a decrease of the merged signals (Yellow), as quantified in Figure 5D, reflecting the re-arrangement in the intracellular localization of these proteins (Figure 5C).

**Figure 6:**
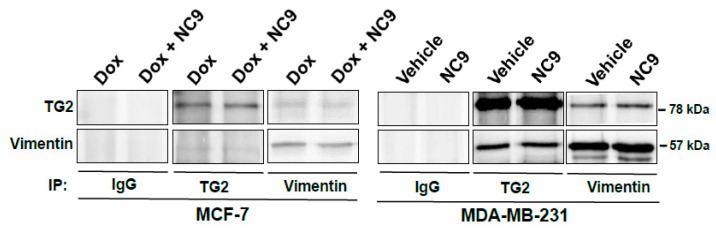
Representative Western blot analysis of TG2 and vimentin in immunoprecipitation from total lysates of MCF-7 treated with Dox and MDA-MB-231 cells in the presence or absence of NC9. Immunoprecipitation (IP) was carried out with specific antibodies against TG2 or vimentin. In addition, the control with a non-specific IgG.

To assess the dependence of the co-localization of TG2 and vimentin on their physical interaction, we performed immunoprecipitation experiments on extracts of MDA-MB-231 and MCF-7 cells treated with Dox that were expressing high levels of these proteins. As shown in Figure 6, immunoprecipitated complexes obtained in vitro with antibodies against TG2 or vimentin revealed an association of both proteins and did not show significant differences between untreated and NC9 treated cells. This indicates their direct physical interaction, independent of TG2 catalytic activity.

Our discovery of a physical connection between TG2 and vimentin confined within the cell engaged in motility control is relevant, especially for the role recently conferred to these two proteins as potential therapeutic targets [27,45,46].

**Figure 7:**
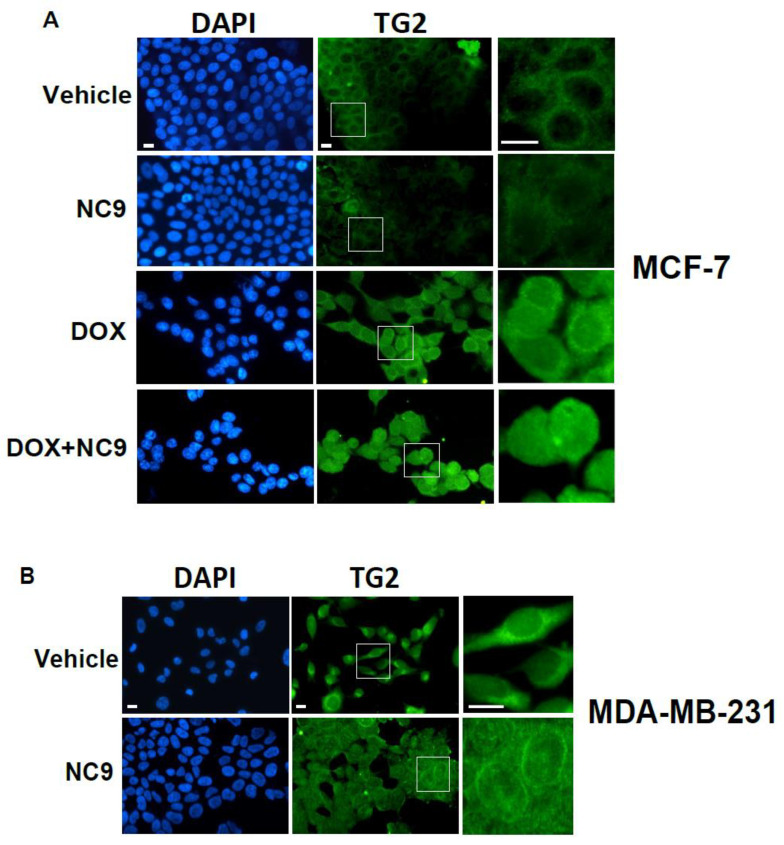

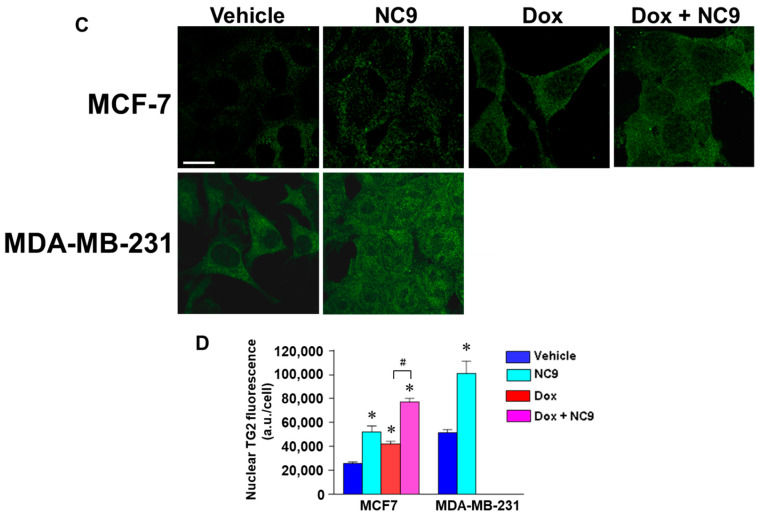
Representative fluorescence images of MCF-7 cells (**A**) grown on glass dishes in the presence of Dox and/or NC9 and (**B**) of MDA-MB-231 cells grown on glass dishes in the presence or not of NC9 subjected to analysis of TG2 with a FITC-coupled antibody. Nuclei were counterstained with DAPI. The regions in the boxes are shown enlarged on the right. (**C**) Representative confocal images of MCF-7 and MDA-MB-231 cells grown under the above reported conditions and subjected to immunocytochemical staining of TG2. (**D**) TG2 nuclear staining, as deduced by analysis of digitized confocal images, under the above reported conditions. Fluorescence values are expressed arbitrary units for cell (a.u./cell) ± SD. Bar = 20 μm. * *p* < 0.05 versus vehicle; # *p* < 0.05 between bars.

A further aspect brought to light by our experiments concerns the subcellular localization of TG2, especially with attention to its nuclear localization. Images of fluorescence microscopy showed that TG2 distribution in MDA-MB-231 cells was analogous to that in MCF-7 cells exposed to Dox (Figure 7A,B) and that NC9 treatment led to an apparent increase in the nuclear content of the protein. The presence of large amounts of TG2 aggregates inside the nuclear compartment was confirmed by confocal microscopy (Figure 7C,D). These data suggest a prominent nuclear role of the protein independent of its enzymatic activity that is possibly implicated in gene expression, as suggested in other reports [47,48].

From this perspective, we have determined that in addition to TG2, the nuclear content of some proteins associated with oncogenesis, cell proliferation, EMT, and invasiveness. We analyzed the amount of several factors engaged with the transcriptional control of MDA-MB-231 cells that were cultured in the absence or presence of NC9. On the basis of evidence emerging both from the literature and from experimental research carried out in our laboratory, we have selected the following proteins: Sp1 and RXR were identified as factors of the MDA-MB-231 regulome implicated in invasiveness [49] and were associated with TG2 expression [50,51,52], while RARα, MAX, and YY1 cooperate with a non-coding RNA expressed by the *TGM2* gene and are involved in the control of a panel of genes that is associated with TG2 activity in MCF-7 cells [53]. Additionally, Eukaryotic translation initiation factor 3 β (EIF3β) was recently found to be strongly associated with EMT via the Akt pathway [54], and as such, c-Myb was controlled by ZEB1 [55]. Finally, we considered GAPDH, a moonlighting protein that also displays nuclear functions because it is a known substrate of TG2 [56].

**Figure 8:**
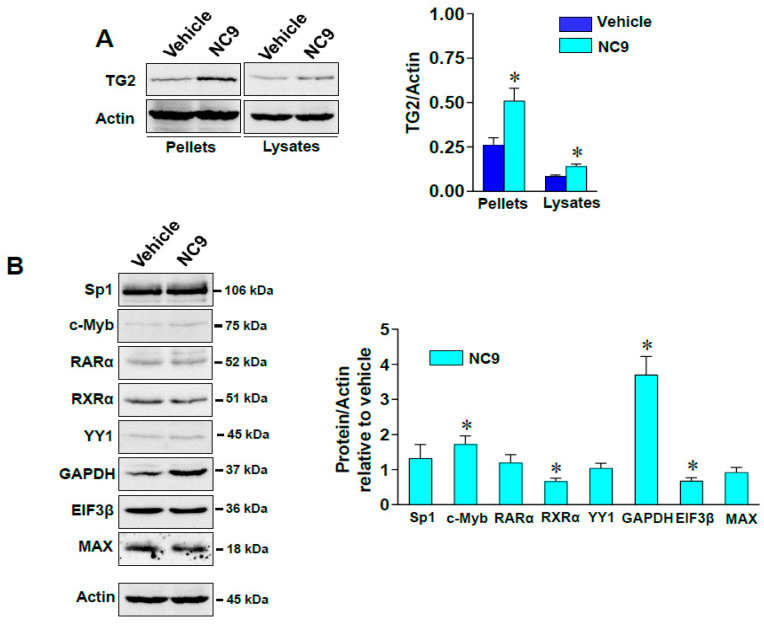
(**A**) Representative immunoblot analysis performed with the indicated antibodies on nuclear pellets and nuclear lysates from MDA-MB-231 cells grown in the presence of vehicle or NC9. On the right are the levels of TG2, as deduced from the analysis of chemiluminescence and normalized with actin, which was used as internal control for equivalence of protein load. (**B**) Representative immunoblot images derived from Western blot analysis with the indicated antibodies of nuclear lysates from MDA-MB-231 cells treated or not with NC9. On the right, levels of the analyzed proteins as deduced from chemiluminescence signals that had been normalized with actin. All values were relative to the vehicle treatment condition, taken as 1. Data are shown as mean of three independent experiments performed in triplicate ± SD. * *p* < 0.05 versus vehicle.

Western blot analysis confirmed an enrichment of TG2 in the nuclear membrane/DNA pellets after treatment as well as in the soluble phase of the nuclear extracts (Figure 8A). Some of the selected factors were modulated after NC9 treatment (Figure 8B). In summary, the levels of TG2 itself, GAPDH, and c-Myb increased, while the amounts of RXRα and EIF3β slightly decreased, suggesting that the TG2 enzyme is also an active modulator of gene expression in invasive BrCa cells [49].

## 4. Discussion

Over the years, several reports have demonstrated multiple roles of TG2 in support of apparently contrasting functions, including cell death and proliferation [57,58], in normal and neoplastic tissues. These opposing effects have largely been ascribed to the bifunctional activities of the enzyme that are involved in both protein cross-linking and in the transduction of extracellular signals, which act as a G-protein.

The polyhedric aspects of TG2 are amplified by additional factors related to its subcellular distribution that are predominantly cytosolic but that are also associated with plasma membranes on the intra- and extracellular side, with the nucleus, and with other intracellular organelles and vesicles [59]. It is therefore conceivable that TG2 plays differential, and in some cases tissue-specific functions [60], in both normal and pathologic conditions. In tumors, including BrCa [1], TG2 is frequently expressed at higher levels than it is in normal tissues and is involved in the maintenance of cancer stem cells, in sensitivity to therapy, in vascularization, and in spreading by metastasis [61].

Assuming that the expression levels of TG2 in the tumor or in surrounding tissues (notably in stroma) [62] correlate positively to a mesenchymal phenotype, promoting EMT as a consequence of drug-resistance [63], in this study, we have analyzed the relationships between TG2 activity and the motility of breast tumor cells. Our results underline intracellular changes rather than the already known extracellular TG2 functions and the possible linkage with another player, vimentin, which regulates the intermediate filaments [24,28,29] and that has also been reported as target of the catalytic activity of TG2 [19,20]. We have focused on MCF-7 cells, which show an epithelial-like phenotype and express TG2 at low levels but that can modify their phenotype into mesenchymal following exposure to Dox [21,22], which induces TG2 expression in association with the phenomena of drug resistance and cell survival [12]; we also included MDA-MB-231 cells in this study because they show high levels of TG2 [64] and represent a mesenchymal-like phenotype that is associated with poor differentiation and high invasiveness. We believe that the increase of TG2 following Dox treatment constitutes an early MCF-7 cell response, appearing 24 h after drug addition. In both cell lines the amount of TG2 correlated with their ability to move, as shown by the reduction promoted by NC9 acting as a TG2 inhibitor, which was also effective when administered to control cells that had not been exposed to Dox.

The evaluation of specific markers of EMT, such as E-cadherin, N-cadherin, and vimentin, in MCF-7 cells confirmed the role of Dox in promoting mesenchymal features, and conversely, the administration of NC9 almost completely blocks the effects of Dox on E-cadherin and N-cadherin, promoting an epithelial phenotype. Concerning vimentin, it was expressed in MCF-7 cells in the presence of Dox, and its expression was only slightly decreased by NC9. These considerations are supported by the results obtained with MDA-MB-231 cells, which show post-EMT features, in which NC9 was unable to consistently modify the expression of vimentin.

Furthermore, we considered the effects of NC9 and the involvement of TG2 by investigating both migration and invasion. In this context, we did not observe any effects of Dox on the invasiveness of MCF-7cells, which is at variance with the results reported previously by Liu et al. [65]. However, it is worth noting that this previous study was carried out under mild conditions, with short exposure to low doses of Dox (treatment at 0.2 μM for 3 h, monitoring for 48 h). Although the relationship between TG2 and invasion has been previously reported for other tumors, such as those found in colon and pancreatic cancer [66,67] and in drug resistant and aggressive BrCa [1], we did not detect any efficacy of NC9 in altering the ability of BrCa cells to migrate through specific matrigel concentrations. The apparent discrepancy of our data compared to the decreased invasion observed by Mangala et al. [23] in MDA-MB-231 clones can be explained by the silencing of TG2 expression, which implies a strong reduction of its levels that does not only affect the catalytic activity of the enzyme, as was the case in our investigations. This suggests that it is not enough to merely block the enzyme, but also that the cellular content influences invasion [68], changing its distribution inside of and outside of the cell, including its interactions with components of the extracellular matrix.

We also focused on the intracellular role of TG2, studying its relationship with vimentin, which constitutes the intermediate filaments in eukaryotic cells. Vimentin is also attached to the nucleus, endoplasmic reticulum, and mitochondria, playing a significant role as support and anchorage of organelles in the cytosol. The action of the vimentin to maintain cell shape and cytoplasmic integrity and to stabilize cytoskeletal interactions makes it a pivotal player impacting the secretion, migration, and invasion properties of cancer cells [18]. A connection between TG2 and vimentin was originally established by the discovery that vimentin is a transglutaminase substrate undergoing cross-linking to stabilize the intracellular filaments [19,20,69,70]. We have now demonstrated that in BrCa cells expressing relatively high levels of TG2 and vimentin, the enzyme is mainly localized in the perinuclear regions, in which vimentin is also present. Experiments of co-immunoprecipitation revealed a physical interaction between these two proteins that was not modified using the TG2 inhibitor and must thus be considered independent of TG2 catalytic activity. The intracellular localization of their interaction seems however to change after treatment with the TG2 inhibitor, as evidenced by the highly expressing MDA-MB-231 cells. Following treatment with NC9, the amount of vimentin decreased. This effect could modify its known interaction with integrin and have an impact on the adhesion capacity [29]. NC9 could affect the in vivo conformation of TG2 [71] and could also likely affect its interactions with the more complex structures of the polymerized filaments of the cytoskeleton, which are essential for the maintenance of precise cellular functions [72].

Two isoforms of the enzyme are present in these BrCa cells; one is full-length, and the other is truncated. Both are increased by Dox in MCF-7 and are constitutively expressed in MDA-MB-231 cells [21,64,73]. The use of the irreversible inhibitor NC9, which locks TG2 in the open form, should affect both variants [74]. In addition, full-length TG2 can assume an open configuration with transamidase activity or the closed form, which exhibits GDP/GTP-binding and GTP hydrolysis in its activity as a G-protein to mediate intracellular signaling, while the truncated isoform only presents the open conformation, which is less active with respect to transamidation and GDP/GTP-binding, but retains high GTPase function [75]. All these activities could be inhibited by NC9. It would be interesting to conduct further investigations using inhibitors that are selective for the closed form (e.g., CP4d) [31,74] or inhibitors that do not modulate conformational changes to compare such results against the results obtained with NC9 that have been reported herein. This would help us to understand if these aspects could be attributed to one specific isoform. It would be important to know if there is an aspect of the observed effects that is also ascribable to the short isoform in order to determine an approach for selective functional modulation.

An additional aspect that emerges from our experiments focuses on the intracellular localization of TG2 in Dox-induced MCF-7 and in MDA-MB-231 cells concerning its nuclear distribution. Through confocal analysis, we demonstrated the accumulation of TG2 inside the nucleus of both cell lines because of NC9 administration, and the appearance of enzyme aggregates are suggestive of nuclear function. In the literature, the involvement of TG2 has been reported in several nuclear processes, including gene expression [4], chromosome condensation [76], and histone modification [19] as well as targeting of several proteins such as ribonucleoproteins [77], which have an impact on the balance of the transport to and from the nucleus [48,78]. In support of nuclear activity, we demonstrated that the use of the TG2 inhibitor NC9 modulated the levels of some transcription factors and regulatory proteins present in nuclear extracts from MDA-MB-231 cells and that are involved in oncogenesis, cell proliferation, and differentiation. Some of these proteins increased (TG2 itself, GAPDH and c-Myb), while others decreased (EIF3β, RXRα), indicating the significance of nuclear modifications and laying the basis to understand the changes of the regulome managed by TG2 in BrCa that are also modified by NC9. On the one hand, an increase of c-Myb might correlate with tumor cell ability to seed metastasis [79] as well as with the transcriptional regulation of epithelial genes in association with EMT in BrCa, while the overexpression of GAPDH in the nucleus occurs during apoptosis [80], and it has also been associated with cancer cell senescence [81] or with DNA repair processes [82]. On the other side, the down-regulation of EIF3β is reported to inhibit cell proliferation and metastasis [83], while the effects of RXRα in the context of BrCa are more complex, owing to their interactions and interference with similar nuclear receptors [84].

In conclusion, the interactions between TG2 and vimentin strongly correlate with the motility of BrCa cells as early events occurring after treatment with the anticancer drug Dox. We think that these effects must be mainly attributed to migration properties, while invasiveness may depend on additional phenotypic/genotypic aspects. In any case, this relationship involves both their intracellular and nuclear localization, suggesting that targeting TG2 [62] could have repercussions not only on motility but that also possibly result in extensive gene expression changes that remain to be defined in greater detail.

## Data Availability

Not applicable.
